# Coccidioidomycosis of the Vocal Cords Presenting in Sepsis: A Case Report and Literature Review

**DOI:** 10.1155/2020/8025391

**Published:** 2020-04-20

**Authors:** Curtis Converse, Atish Dey, Samuel Decker, Sarkis Arabian, Michael Neeki

**Affiliations:** ^1^Department of Internal Medicine, Arrowhead Regional Medical Center, Colton, CA, USA; ^2^Department of Emergency Medicine, Arrowhead Regional Medical Center, Colton, CA, USA

## Abstract

Coccidioidomycosis is a predominantly pulmonary disease caused by species of *Coccidioides*, a fungus endemic to the American Southwest. Most cases involve exclusively pulmonary manifestations while less than one percent present with disseminated infection, usually with meningeal or skin involvement. In this case, a patient with a history of odynophagia, sore throat, productive cough, weight loss, and abnormalities on chest radiograph presented with sepsis and diabetic ketoacidosis. During admission, the patient underwent bronchoscopy with resulting tissue and bronchoalveolar lavage samples positive for *Coccidioides immitis*, later supported by confirmatory serum studies. This case illustrates a rare presentation of vocal fold involvement without direct invasion from a continuous site and highlights the importance of a high index of suspicion for disseminated coccidioidomycosis with prompt antifungal treatment in order to avoid the very high morbidity and mortality in such cases.

## 1. Introduction

Coccidioidomycosis is a fungal disease that may cause pulmonary or disseminated infection [1, 2]. The distributions of the causative dimorphic fungi, *Coccidioides immitis and C. posadasii*, are overlapping and largely confined to the Southwestern United States, Northern Mexico, and Central America, with sporadic endemic cases also reported in Washington State [1–3]. They exist in the environment as molds with septated hyphae which contain the infectious arthroconidia. When the dry soil in which the fungus dwells is disturbed, the arthroconidia become airborne and are inhaled. Inside a host, at temperatures of 37-4°C, the mold matures to form spherules filled with endospores, which then cause local infection in 99% of cases or dissemination in <1% of the cases. It is estimated that over half of coccidioidomycosis cases are asymptomatic or minimally symptomatic and self-limited. Those who do develop pulmonary symptoms usually have complaints lasting several weeks. Respiratory symptoms include nonproductive cough, coryza, and pleuritic or dull chest pain [3]. Dissemination is most likely to occur in those of African or Filipino descent, at the extremes of age, with diabetes, pregnant, immunosuppressed, and in a newly recognized group with genetic mutations in genes related to interferon-gamma and interleukin 12 [2, 4]. The immune reaction is typically marked by caseating granulomas or pyogenic reaction, which may be misdiagnosed and mistreated as either chronic granulomatous disease or bacterial infection with abscess. Interestingly, most cases are diagnosed in winter [1]. Here, we present a rare case of coccidioidomycosis of the vocal cords in a patient complaining of chronic dysphonia or dysphagia.

## 2. Case Presentation

A 52-year-old male presented to the emergency department with four days of sore throat, odynophagia, and productive cough with clear-white, blood-tinged sputum. He also noted unintentional weight loss of approximately 10 pounds over the past three months but denied dyspnea, night sweats, fever, chills, or prior pulmonary complaints. Additional complaints included nausea, vomiting, polydipsia, and polyuria for the past few days after he stopped taking his long-acting insulin to adjust for decreased oral intake. He was sent to the emergency department by his primary care physician for abnormalities on chest X-ray taken the previous day, at which time he had been prescribed cephalexin empirically for suspected community acquired pneumonia.

His past medical history was significant for type 2 diabetes mellitus controlled with metformin and long-acting insulin without previous hospitalizations. He immigrated to the United States approximately 40 years ago from Central America and had never left the United States since. He works as short-distance truck driver and previously smoked heavily for a decade before quitting 20 years ago.

Upon arrival to the emergency department, his vital signs included blood pressure 123/90 mmHg, heart rate 134 beats per minute, respiratory rate 40 breaths per minute, temperature 97.8°F, and oxygen saturation 98% on room air. His physical exam revealed a cachectic man in mild distress with tachypnea, without nasal flaring or retractions, with dry mucous membranes, and hoarse voice. Lung auscultation revealed diffuse bilateral crackles. Cardiac exam revealed tachycardia with regular rhythm. Abdominal exam did not demonstrate hepatosplenomegaly or tenderness. There were no other significant physical findings on examination.

**Figure 1:**
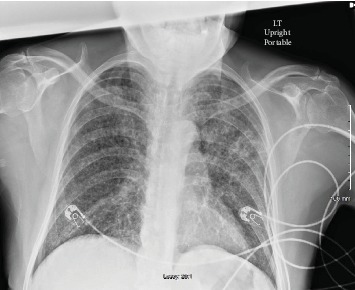
Chest radiograph upon arrival to the emergency department revealed diffuse bilateral reticulonodular opacities and airspace disease with a possible lingular cavitary lesion.

**Figure 2:**
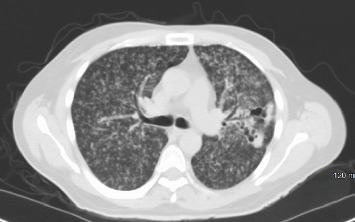
Thoracic CT scan without contrast demonstrating numerous, bilateral tiny nodular densities, most prominently in the upper lobes and confirmed a lingular cavitary lesion.

His chest X-ray revealed diffuse bilateral reticulonodular opacities and airspace disease with a possible lingular cavitary lesion (Figure 1). A thoracic CT without contrast was obtained which revealed numerous, bilateral tiny nodular densities, most prominently in the upper lobes, and confirmed the lingular cavitary lesion (Figure 2). A few scattered mediastinal lymph nodes were also noted. Serum chemistry was remarkable for an anion gap of 35, beta-hydroxybutyrate of 8.09 mmol/L, and blood glucose of 532 mg/dL. Hematology showed a white blood cell count of 13 000/*μ*L with 0% eosinophils. Venous blood gas analysis revealed a pH of 7.13, pCO2 of 32, and lactic acid of 5.46 mmol/L. Urinalysis was positive for 4+ glucose and 3+ acetone. Later, HIV antigen testing for types 1 and 2 was negative and CD4 count was 343 cells/*μ*L. Vancomycin 1 g and piperacillin/tazobactam 3.375 g were given intravenously in the emergency department. Intravenous insulin was started with dose adjustment according to an in-house nursing algorithm alongside vigorous hydration.

He was admitted to the ICU on airborne isolation with differential diagnoses including coccidioidomycosis, tuberculosis, and lymphangitic carcinomatosis, complicated by diabetic ketoacidosis. In the ICU, empiric antifungal therapy with fluconazole 800 mg/day was started intravenously for suspected pulmonary Coccidioides infection. Although serum anion gap and bicarbonate normalized on day two, he remained tachycardic and tachypneic and required supplemental oxygen. He also continued to have odynophagia that necessitated enteral tube feeding until hospital day five, when he was finally able to tolerate an oral diet. At this point, he developed a mild elevation of transaminases and fluconazole was changed to itraconazole 200 mg twice daily due to concern for hepatotoxicity, and abdominal ultrasound and computed tomography (CT) were obtained to rule out hepatic involvement from the suspected fungal infection. Both of these studies were negative for ascites, focal hypoattenuation within the liver, or hepatic or splenic enlargement. The CT additionally did not demonstrate abdominal adenopathy. Aminotransferases trended from their peak on hospital day three to normal on day eight.

A week after admission, he remained tachypneic, hypoxic, and hoarse. Three sputum samples were negative for acid-fast bacilli but Coccidioides antibody titer and complement fixation had not yet resulted; thus, bronchoscopy was performed, revealing irregular-appearing borders of false and true vocal cords with erythema and edema, two vocal cord polyps, inflamed upper trachea in the subglottic space, and several flesh-colored endobronchial lesions bilaterally. Forceps biopsies and brushings were performed on the endobronchial lesions, true and false cords, and posterior trachea just below the larynx. Bronchoalveolar lavage (BAL) was performed in the right middle lobe and lingula. BAL fluid cell count and differential showed a neutrophilic predominance with spherules and endospores, consistent with *Coccidioides immitis* or *Rhinosporidium seeberi*. The biopsy specimens demonstrated the same pathogen in the laryngeal tissue. The culture of the BAL fluid eventually grew *Coccidioides immitis* on the day prior to discharge (day 14), but the laryngeal tissue was not cultured.

Otolaryngology consultant recommended outpatient follow-up with consideration for laryngoscopy after resolution of his acute illness. Two days after bronchoscopy, Coccidioides antibody titer returned positive at 1 : 16. The infectious disease consultant recommended continuing itraconazole for a minimum of 12 months with serial monitoring of Coccidioides titer, transaminases, and itraconazole level every three months. He continually improved on antifungal therapy until successfully weaning from supplemental oxygen and discharge after two weeks of hospitalization.

### 2.1. Follow-Up

Coccidioides complement fixation returned strongly positive at 1 : 1024 nine days after discharge. Histoplasma urine antigen was positive but below the limit of quantification. At his follow-up appointments two and twelve weeks after discharge, his productive cough was present but improved and his hoarseness, dyspnea, and odynophagia had resolved. His liver enzymes remain within normal limits. At time of writing, the patient had not yet returned to have his Coccidioides titer or itraconazole level measured.

## 3. Discussion

It is estimated that over half of coccidioidomycosis cases are asymptomatic, minimally symptomatic, or self-limited. Those who develop pulmonary symptoms usually have complaints lasting several weeks. These include nonproductive cough, coryza, and pleuritic or dull chest pain. Common systemic symptoms include fever, headache, fatigue, and malaise. Due to the similar presentations, a majority of patients are treated with antibiotics for suspected bacterial pneumonia before eventually being correctly diagnosed [4]. There are also common extrapulmonary, immune-mediated manifestations such as mono- or oligoarthritis, especially of the knee, erythema nodosum, and erythema multiforme. These are distinct from extrapulmonary infection and give rise to the epithet, “Desert Rheumatism.” The pulmonary disease can progress to cavitary pneumonia, especially of the upper lobes, and pyo- or pyopneumothorax [1]. Though rare, sepsis due to Coccidioides does occur and is associated with a mortality rate near 100% [4].

Our review of the published literature does not reveal any cases of isolated laryngeal involvement, but several cases of secondary involvement have been reported. It is unclear whether these always represent inoculation from direct contact with the infectious source or if there is a possibility of hematogenous spread to the larynx [3, 5, 6]. See Table 1 for a summary of all reported cases of laryngeal coccidioidomycosis.

Several cases of prevertebral and retropharyngeal abscesses with extension to the larynx have been reported [7–10], but very few have had vocal fold involvement without invasion from contiguous structures [5, 10–14]. Those that do not have direct extension have had concomitant involvement of another extrapulmonary site, namely, axial skeleton or skin. Laryngeal symptoms include hoarseness, sore throat, dysphagia, and odynophagia. Several of the cases previously mentioned were initially suspected to be cancerous due to their appearance on laryngoscopy before biopsy showed otherwise [5–7, 14].

Imaging of the neck in cases of laryngeal coccidioidomycosis without direct extension from another site is most often normal. In fact, only one case showed any abnormality on CT of the neck, specifically with supraglottic narrowing due to edema [5]. It should be borne in mind, however, that several of the reported cases occurred before the wide availability of CT. In cases of retropharyngeal or prevertebral abscesses, there is occasionally visible compromise of the airway by external compression.

Due to the paucity of cases of laryngeal Coccidioides, there have been large lapses in time between cases and appropriate treatment leading to substantial advances in antimicrobial therapy in the intervals. The earliest cases were treated with iodides and radiation [11] or dihydroxystilbamidine [12], followed by amphotericin B and the azole antifungals, as each agent was successively discovered over the following decades [15].

Duration of therapy for these cases has similarly evolved, with early cases treated for shorter duration than more recent cases. As several of the reported cases have been in patients with previous diagnoses of disseminated disease [5, 14], patients may end up on life-long suppressive therapy. Two cases have been reported in patients receiving immunosuppressive therapy. One of these involved a child who experienced two episodes of disseminations, once while on cyclophosphamide and prednisone for chronic granulomatous disease and again after receiving kidney transplant with further immunosuppression [16, 17]. The other case occurred in a patient taking steroids chronically for bronchiectasis [14].

The current guidelines of Infectious Disease Society of America recommend treatment of disseminated coccidioidomycosis with fluconazole 400-1200 mg/day, but do not recommend a specific duration or endpoint. The exception to this is coccidioidal meningitis, which necessitates life-long suppressive therapy regardless of symptoms. Amphotericin should be used in cases of severe or life-threatening infection or if there is treatment failure with azole therapy [2].

While any case of vocal fold coccidioidomycosis can be considered unique, there are some aspects of this case that adds to our scant literature about this subject. Firstly, this is the only reported case with concurrent diabetic ketoacidosis, despite the fact that it is known that diabetes is a risk factor for complicated pulmonary disease. Secondly, data from literature indicated that only about quarter of all sputum or BAL samples from patients with active pulmonary coccidioidomycosis culture are positive [3]. However, it is estimated that 100% of those with active pulmonary disease and sepsis or septic shock are culture positive from respiratory sources [18]. Finally, the majority of the previously reported cases had either previous pulmonary coccidioidomycosis [6, 13, 14] or other signs of disseminated disease to the skin [6, 12], meninges [5], or bone [6, 12], none of which was seen in this case.

With regard to the treatment rendered in this case, the patient was started empirically on antifungal therapy, initially with moderate-dose fluconazole 800 mg/day, which is midrange for the IDSA recommendation of 400-1200 mg/day, and transitioned to itraconazole 200 mg twice daily because of concern for developing hepatotoxicity. This change was likely premature, as the elevation in aminotransferases was mild (<2 times the upper limit of normal) and was not trended to determine if it was worsening, but the change did not appear to impact the efficacy of the treatment. Evaluation for liver disease was performed but did not demonstrate any abnormalities. Findings that would have been consistent with the diagnosis of hepatic Coccidioides include focal hypoattenuation, ascites, hepatosplenomegaly, and abdominal adenopathy [19].

The reason for starting early treatment with antifungal therapy empirically when it was only one of three major differential diagnoses (the others being mycobacterial infection and lymphangitic carcinomatosis) was based on the acuity of his presentation. While tuberculosis was not ruled out until several days after admission, sepsis and acute respiratory failure are uncommon presentations of active TB and would be unlikely to respond quickly to antibiotics even if they were administered rapidly [20, 21]. Furthermore, the disease is endemic in our area, with 85 cases reported in our county in 2017, the most recent year for which data is available [22].

He was kept in airborne isolation until the required three negative acid-fast smears were resulted. Due to the severe presentation of his disease, he was ruled out for HIV infection but did demonstrate a low CD4+ lymphocyte count. This is likely due to the fungal infection itself rather than the cause of the infection [23]. The positivity of the Histoplasma antigen is not unexpected, as there is significant cross-reactivity between Histoplasma and Coccidioides antigens, nearly 80% in one review [24]. Another interesting point is the level of antigen titer. Previous studies have demonstrated that reactivity of greater than or equal to 1 : 16 is frequently indicative of disseminated infection, which is consistent with our patient [2].

## 4. Conclusion

This case highlights several important points about coccidioidomycosis, a common disease in the Southwest that appears to be on the rise. The expanding endemic region of the fungus and the increased ability to travel through endemic regions over recent decades should encourage physicians outside of the historic geographic zone to be familiar with the disease.

Due to the extreme variety of symptoms that can be caused by Coccidioides infection, clinicians should have a high index of suspicion for fungal pneumonia if the patient presents with atypical or nonresolving symptoms, especially if those symptoms are extrapulmonary. Hoarseness may be an extremely rare symptom of infection of the vocal cords in patients with pulmonary disease suspicious for coccidioidomycosis. In patients with coccidioidomycosis presenting with sepsis, early initiation of antifungal therapy may decrease mortality, contrary to previous reports.

## Figures and Tables

**Table 1:** Reported cases of laryngeal coccidioidomycosis without contiguous spread (7, 10, 11, 12, 5, 6, 13, 14, 3).

Age/sex	Symptoms	Laryngeal diagnosis	Other sites	Treatment	Outcome
40/F [13]	Hoarseness, lymphadenopathy, fever, night sweats, dry cough, fatigue	Edematous and erythematous mucosa without focal lesions	Lymphatics, pulmonary	Fluconazole	Improvement
52/F [14]	Dysphonia, cough, weight loss, odynophagia, dysphagia	Epiglottic, aryepiglottic, and piriform fossa erythema, edema; vocal fold paralysis	Pulmonary	Fluconazole 400 mg twice daily for 6 weeks, then extended	Resolution of symptoms, left vocal fold paralysis
2.6, 14/M [14]	Not reported	Laryngeal infection on immunosuppressants, recurrence after 12 year	Not reported	“Systemic antifungal treatment”	Remission
37/M [11]	Odynophagia, dysphagia, hoarseness, night sweats, hemoptysis	Coccidioidal granuloma of the epiglottis	Lymphatic	Laryngeal irradiation and iodides for 3 months	Discharge to “full employment”
34/M [12]	Productive cough ×3 months, hoarseness, dysphagia ×1 month, weight loss	Fungating granuloma of endolarynx	Bone, pulmonary, skin	Tracheostomy, dihydroxystilbamidine 150 mg IV 4 times daily for 20 doses	None reported
45/M [6]	Productive cough, fever, malaise, hemoptysis, weight loss, dyspnea, headache, hoarseness	Aryepiglottic granuloma	Pulmonary, bone marrow	Amphotericin B 2 g, then miconazole nitrate	Laryngeal lesions cleared, disseminated infection after 3 years
34/M [5]	Not reported	Epiglottic erosion, “heaped up involvement” of entire endolarynx	Pulmonary	Tracheostomy	Not reported
20/F [5]	Not reported	Granulomatous appearance of posterior commissure, false cords, aryepiglottic folds	Meninges	Amphotericin B, parenteral and weekly intrathecal	Not reported
“Adult” age/sex not given [5]	Not reported	Granulomatous appearance of posterior commissure, false cords, aryepiglottic folds	None	Not reported	Not reported
19 mo./sex not given [5]	Not reported	Obstructive granuloma of the trachea and larynx	Pulmonary	Not reported	Not reported
4.5 mo./M [5]	Wheezing, cough	Obstructive subglottic and anterior commissure granuloma	Pulmonary	Tracheostomy, amphotericin B 0.1 mg/kg/day increasing to 1 mg/kg/day to a total of 271 mg	Improvement
